# Identifying optimal candidates for primary tumor surgery in patients with metastatic head and neck cancer

**DOI:** 10.3389/fsurg.2024.1394809

**Published:** 2024-04-10

**Authors:** Qi-Wei Liang, Shuang-Hao Zhuang, Sheng Li

**Affiliations:** ^1^ Department of Otorhinolaryngology of Longgang Center Hospital, The Ninth People’s Hospital of Shenzhen, Shenzhen, China; ^2^ Department of Otorhinolaryngology Head and Neck Surgery, Department of Thyroid Center/Thyroid Surgery, The Sixth Affiliated Hospital of Sun Yat-sen University, Guangzhou, China

**Keywords:** metastasis, predictive models, nomogram, head and neck cancer, SEER

## Abstract

**Background:**

Primary tumor surgery (PTS) may enhance survival among part of patients with metastatic head and neck cancer (mHNC). Herein, a predictive model was needed to construct to identify who can gain benefit remarkably from tumor resection.

**Methods:**

Data of patients with mHNC were extracted from the Surveillance, Epidemiology, and End Results (SEER) database. The best cut-off value of age were analyzed using the X-tile software. One-to-one PSM, Kaplan–Meier method, and log-rank test were performed for survival analysis.The independent factors determined using the multivariate Cox proportional hazard regression were used to construct the nomogram.

**Results:**

A total of 1,614 patients diagnosed with mHNC were included; among them, 356 (22.0%) underwent a surgical procedure for the excision of the primary tumor. cancer-specific survival (CSS) was remarkably prolonged in the PTS group relative to the non-PTS group following PSM [Median:19 months vs. 9 months; hazard ratio (HR) 0.52, *P* < 0.001]. Patients with mHNC who were younger than 52 years old, had well-differentiated tumors, had T1 and N0 stages, and were married at the time of the study may have significantly benefited from PTS. In addition, we constructed a nomogram based on the factors that independently affect the CSS in multivariate Cox analysis. The nomogram showed excellent discrimination in both the training and validation sets (AUC: 0.732 and 0.738, respectively).

**Conclusion:**

A practical predictive model was constructed to determine the appropriate patients with mHNC, who would benefit from surgical resection.

## Introduction

Head and neck cancer (HNC) is the sixth most prevalent malignancy, globally, with squamous cell carcinoma being the most prevalent histological type (accounting for approximately 90%) (1). The distant metastasis rate in HNC ranges between 3% and 20% (2, 3), whereas the median survival time is approximately 6–10 months (4, 5). For metastatic head and neck cancer (mHNC) patients, systemic therapeutic interventions, including targeted therapy, chemotherapy, optimal supportive therapy, and local treatments, such as radiotherapy, are the conventional procedures at present. Although the studies on chemotherapeutic regimens have achieved significant progress in the past few years, the improvement in survival time is relatively small (6, 7) and the high rates of toxicities are common (8). In recent years, immunotherapy has also made good progress, especially the use of immune checkpoint inhibitors (9, 10). However, what is worrying is that the incidence of hyperprogression of head and neck squamous cell carcinoma is relatively high, up to 29%, and is significantly associated with a poor prognosis (11).

Primary tumor surgery (PTS) is also one of the methods for the treatment of mHNC. The 2023 National Integrated Cancer Network (NCCN) guidelines on HNC which can obtained from the NCCN website (www.NCCN.org) endorse regional treatment for mHNC patients, while PTS is still a relatively rare option (12). Indeed, several recent studies show that surgery may significantly prolong the survival time in patients with mHNC (13, 14). This may be partly because surgery has improved the basic functions of the human body, including breathing and swallowing, which in turn affects the survival of patients (15). In addition, PTS reduces tumor load and complications, making patients more likely to benefit from multimodal treatment, including immunotherapy and chemotherapy (16–18). Therefore, as for mHNC patient, local resection may be useful. Nevertheless, given its association with severe trauma and postoperative complications, not all patients may have prolonged survival duration post-operatively. However, it remains unclear regarding the patients who may benefit most from PTS. Therefore, the individualized prediction model will help clinicians to apply individualized surgical treatment.

Hence, to address the clinical needs, the present study focused on developing a predictive model based on the characteristics and prognoses to identify mHNC patients who could benefit from PTS.

## Methods

### Patient inclusion

The Surveillance, Epidemiology, and End Results (SEER) database is a publicly available cancer repository consisting of information on 28% of the population of the United States [Practical Guide to Surgical Data Sets: Surveillance, Epidemiology, and End Results (SEER) Database]. With the aid of the SEER*Stat software (version: 8.3.9), we obtained data from the SEER repository. Between 2004 and 2015, patients with cancer of the hypopharynx, larynx, oral cavity, or oropharynx who also developed distant metastases from the initial diagnosis were included in the present research (including distant lymph nodes metastasis and organ metastasis). Clinical features were extracted from the database, including information on sex, race, age, pathological category, marital status, TNM staging, pathological grading, and treatment regimens (surgery, radiotherapy, or chemotherapy). Marital status was classified into married and single (including single, divorced, widowed, separated, or domestic partner) groups. Exclusion criteria were: age <18 years, unknown date of diagnosis or death, incomplete survival data and/or follow-up information, and unknown primary site records, neither for initial nor for the only tumor. PTS in the present research was described as the surgical procedures directed to tumors located at the primary site, including total, hemi, or partial glossectomy, subtotal or total laryngectomy, and pharyngectomy; laser ablation (surgery codes 20–28), photodynamic therapy, electrocautery, biopsy, local tumor excision, cryosurgery, and/or local tumor destruction (surgery codes 10–15), were excluded. Cancer-specific survival (CSS) was defined as the period from the date of diagnosis to the date of death due to mHNC.

### Statistical analysis

In accordance with the treatment modality of the primary tumor, the included patients were classified into two groups, namely the PTS group and the non-PTS group. One-to-one propensity score matching (PSM) was used to minimize selection deviation and confusion. A score of the standard deviation less than 10% was determined as an appropriate balancing criterion in the present research (19). The variables used for PSM included sex, race, age, marital status, primary site, TNM staging, pathological grading, radiotherapy, chemotherapy and surgery to distant site. X-tile (version 3.6.1) was used to analyze the cutoff values for the age (20). CSS rate in the two groups were derived by the Kaplan–Meier method and significant survival differences were computed by performing the log-rank test. The independent predictive variables were determined by performing the multivariate Cox proportional hazard regression analysis. In the multivariate Cox proportional hazard regression analysis, variables with *p*-value less than 0.05 are considered to be independent predictors of the prognosis of mHNC patients. These screened variables will be incorporated into the prediction model. Herein, the hazard ratios (HRs) were computed with 95% confidence intervals (CIs). Two-tailed *p* < 0.05 was established as the criterion of statistical significance. All analyses of statistical data were conducted with the aid of R (version 3.6.0) (http://www.r-project.org/). The main outcome is cancer-specific survival.

### Construction and validation of the nomogram

According to the median CSS, we reasonably hypothesized that patients in the PTS group would survive longer relative to those in the no-PTS group and thus benefit more from the surgical intervention. The patients in the PTS group were classified into two subgroups based on this assertion as follows: a surgery-beneficial group (median CSS >9 months) and a surgery-non-beneficial group (median CSS ≤9 months). Moreover, the patients in the PTS group were classified into the training and the validation sets at random in the ratio of 7:3.

The factors affecting CSS were included in the training set based on the results of multivariate Cox analysis. Subsequently, a nomogram was constructed for the purpose of identifying the mHNC patients with a greater likelihood of benefiting from PTS in the training group. The prognostic nomogram was used to calculate the benefit probability for patients undergoing the operative procedure. A prediction probability of >0.5 was considered as a surgery-beneficial candidate, and ≤0.5 was a surgery-non-beneficial candidate.

The area under the ROC curve (AUC) and the calibration map (*p* > 0.05 illustrated that the theoretical correction error was not significant) was used to examine the discriminant ability and performance of the prediction model, respectively. The Kaplan-Meier analysis was used for the purpose of investigating if the prediction model was capable of discriminating between individuals who may gain from PTS and those who would not.

## Results

### Features of patients before and after PSM

**Figure 1:**
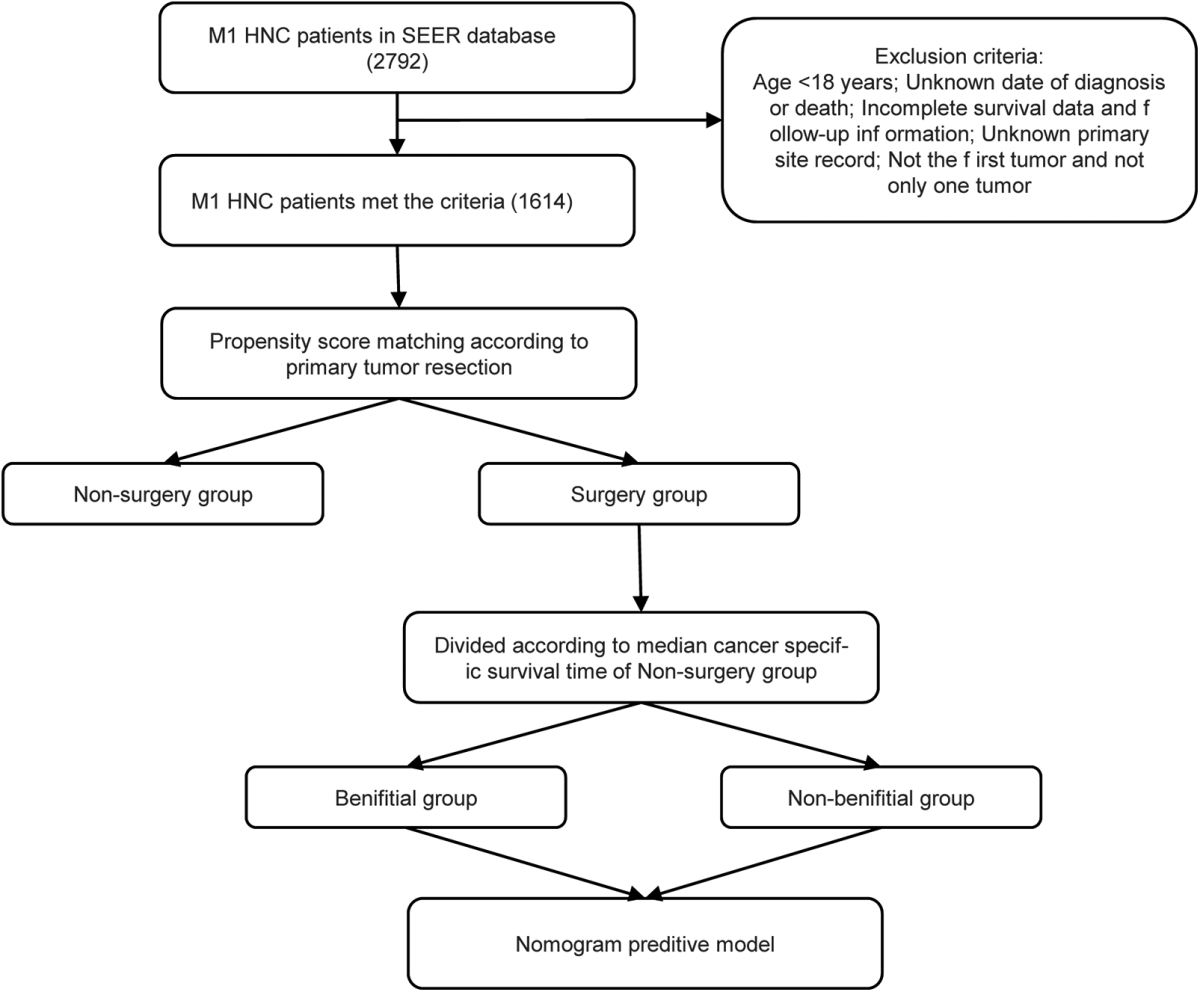
The flowchart for the construction of the prediction model. SEER, Surveillance, Epidemiology and End Results; PSM, propensity score matching.

**Figure 2:**
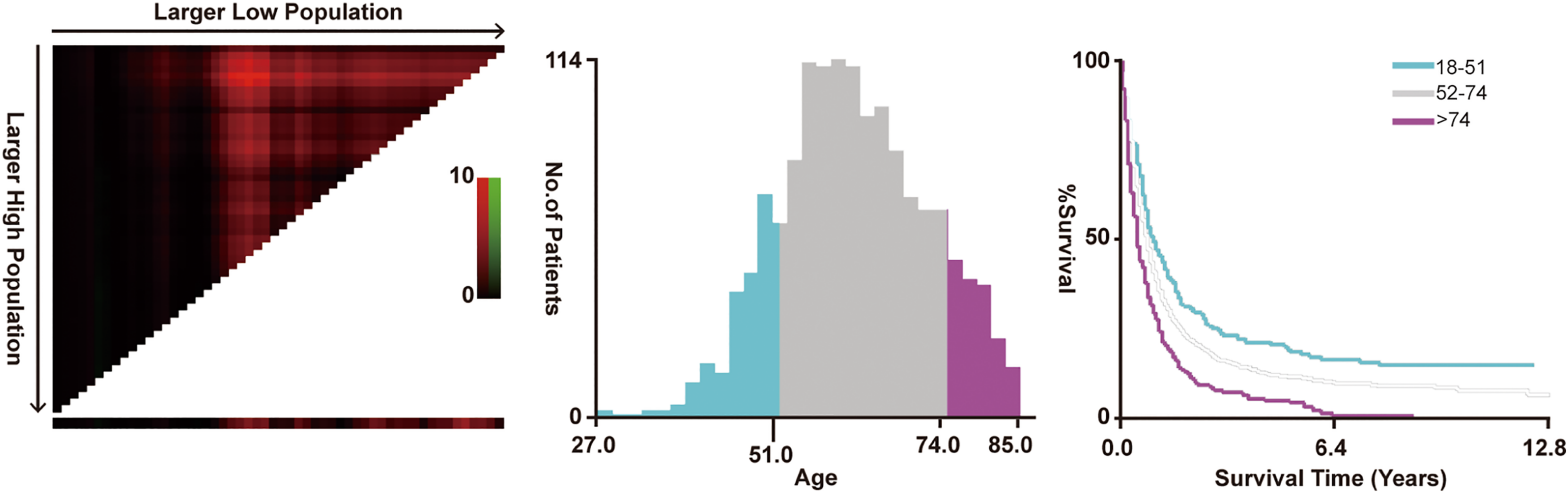
X-tile analysis for disease-specific survival according to age.

The screening of the SEER database yielded a total of 1,614 mHNC patients; among them, 356 (22.0%) underwent PTS. Figure 1 shows the selection process of the present research. A total of 482 patients of mHNC patients treated with or without PTS were enrolled in the study. The threshold value of age was calculated using the X-tile (Figure 2). Table 1 shows the baseline features for the two groups (surgery and no-surgery) before and after PSM. Before PSM, significant differences in variables, including histology, race, primary site, TN stage, histology, marital status, surgery, chemotherapy, and radiotherapy for distant sites, were observed between the two groups, suggesting the absence of balance in the baseline features for the two subgroups. All baseline parameters were well balanced using the 1:1 PSM.

**Table 1:** Characteristics for study population by study groups before and after PSM.

Characteristic	Before PSM			After PSM		
	Surgery to primary site (*n* = 356)	Non-surgery to primary site (*n* = 1,258)	*p*	Surgery to primary site (*n* = 341)	Non-surgery to primary site (*n* = 341)	*p*
Age [mean (SD)]	60.1 ± 11.4	62.9 ± 10.7	<0.001	61.2 ± 10.4	61.9 ± 10.7	0.621
Race (%)						
White	290 (81.5)	933 (74.2)	0.002	284 (83.4)	276 (80.9)	0.141
Black	40 (11.2)	265 (21.1)		36 (10.6)	52 (15.2)	
Other	26 (7.3)	60 (4.6)		21 (6.0)	13 (3.9)	
Gender (%)						
Male	273 (76.8)	1,016 (80.8)	0.062	262 (76.8)	273 (80.1)	0.438
Female	83 (23.2)	242 (19.2)		79 (23.2)	68 (19.9)	
T stage (%)						
T1	61 (17.2)	106 (8.4)	<0.001	56 (16.6)	45 (13.7)	0.768
T2	89 (25.0)	337 (26.8)		83 (24.5)	89 (26.1)	
T3	54 (15.2)	243 (19.3)		52 (15.4)	59 (17.4)	
T4	152 (42.6)	572 (45.5)		150 (43.5)	148 (42.7)	
N stage (%)						
N0	81 (22.7)	168 (13.4)	0.002	72 (21.1)	61 (17.9)	0.42
N1	71 (19.9)	248 (19.7)		69 (20.2)	79 (23.2)	
N2	176 (49.6)	725 (57.6)		170 (49.9)	171 (50.1)	
N3	28 (7.8)	117 (9.3)		30 (8.8)	30 (8.8)	
Primary site (%)						
Oral	183 (51.4)	436 (34.7)	<0.001	171 (50.1)	165 (43.6)	0.072
Oropharynx	86 (24.2)	302 (24.0)		78 (22.9)	88 (27.4)	
Hypopharynx	10 (2.8)	99 (7.9)		10 (2.9)	16 (4.7)	
Larynx	77 (21.6)	421 (33.5)		82 (24.1)	82 (24.1)	
Grade (%)						
I/II	183 (51.6)	641 (51.0)	0.913	170 (49.9)	168 (49.3)	0.927
III/IV	173 (48.4)	617 (49.0)		171 (50.1)	173 (50.7)	
Histology (%)						
Squamous cell carcinoma	320 (89.8)	1,190 (94.6)	0.006	309 (90.6)	208 (90.3)	0.997
Other	36 (10.2)	68 (5.4)		32 (9.4)	33 (9.7)	
Marital status (%)						
Married	192 (53.9)	477 (37.9)	<0.001	182 (53.5)	192 (56.4)	0.583
Single	164 (46.1)	781 (62.1)	** **	159 (46.5)	149 (43.6)	
Radiation (%)						
None/unknown	135 (37.9)	538 (42.8)	0.17	131 (38.6)	126 (36.9)	0.778
Yes	221 (62.1)	720 (57.2)	** **	210 (61.4)	215 (63.1)	
Chemotherapy (%)						
None/unknown	163 (45.7)	448 (35.6)	0.003	150 (44.0)	145 (42.7)	0.854
Yes	193 (54.3)	810 (64.4)	** **	191 (56.0)	196 (57.3)	
Surgery to distant site (%)						
None/unknown	296 (83.2)	1,219 (96.9)	<0.001	300 (88.0)	298 (87.3)	0.683
Yes	60 (16.8)	39 (3.1)	** **	41 (12.0)	43 (12.7)	

PSM, propensity-score matching; SD, standard deviation.

### The impact of PTS on survival in mHNC

**Figure 3:**
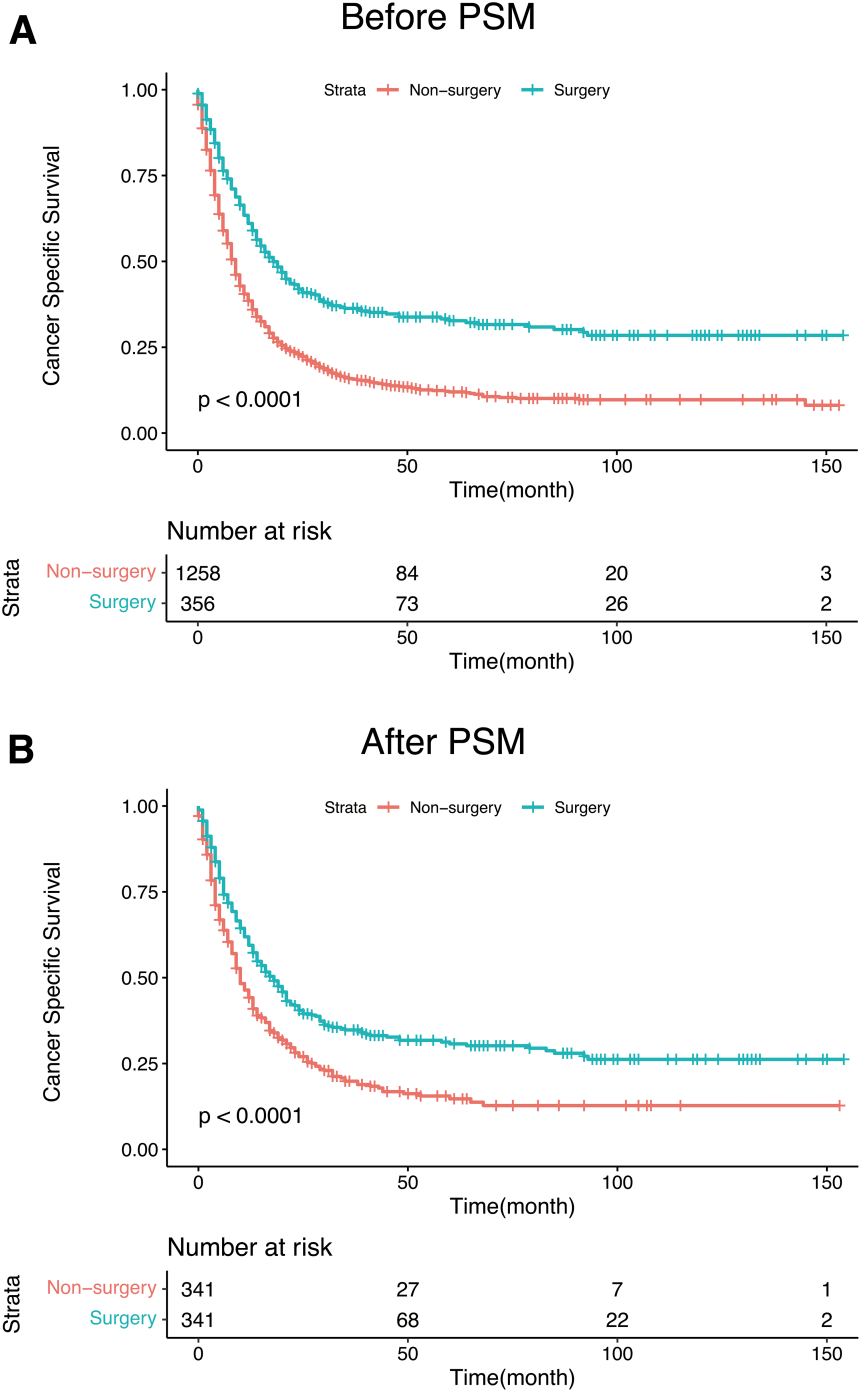
(**A**) Kaplan–Meier curves for CSS of mHNC patients with or without PTS before PSM. (**B**) Kaplan–Meier curves for CSS of mHNC patients with or without PTS after PSM. PSM, propensity score matching; CSS, disease-specific survival; PTS, primary tumor surgery.

Statistically significant differences in survival outcomes between the two groups were observed according to the results of the Kaplan-Meier analysis and log-rank test in the corresponding groups. The PTS group had a better prognosis than the non-PTS group before(20.0 vs. 8.0 months; *p* < 0.001) and after PSM (18 months vs. 9 months; *p* < 0.001) (Figure 3). The results depicted in Table 2 further verified that PTS was independently correlated with the improvement in CSS (HR 0.54; 95% CI, 0.43–0.67, *p* < 0.001). In the multivariate Cox analysis, variables with *p*-value less than 0.05 are considered to be independent predictors of the prognosis of mHNC patients, including T stage, N stage, marital status, age, primary site, surgery, chemotherapy, and radiotherapy.

**Table 2:** Multivariate Cox analysis for CSS before and after PSM in mHNC patients.

Variable	Before PSM			After PSM		
	Adjust HR	95%CI	*P*-value	Adjust HR	95%CI	*P*-value
Age						
18–51	reference			reference		
52–74	1.21	1.12–2.01	0.033	1.19	1.06–1.58	0.266
>74	1.53	1.23–2.63	0.046	1.87	1.32–1.92	0.006
Gender						
Male	reference			reference		
Female	1.04	0.80–1.33	0.782	1.12	0.86–1.42	0.435
Race						
White	reference			reference		
Black	1.12	0.84–1.5	0.451	1.08	0.79–1.48	0.626
Others/unknown	1.05	0.63–1.73	0.859	1.01	0.59–1.73	0.969
Marital status						
Married	reference			reference		
Single	1.34	1.09–1.66	0.006	1.52	1.2–1.93	<0.001
Primary site						
Oral	reference			reference		
Hypopharyngeal	1.17	1.04–1.48	0.027	1.22	1.05–1.56	<0.001
PrimaryLaryngeal	0.94	0.73–1.22	0.658	0.88	0.70–1.39	0.172
Oropharyngeal	0.90	0.68–1.18	0.441	0.78	0.52–0.94	0.017
Grade						
Grade Ⅰ/II	reference					
Grade Ⅲ/V	1.16	0.94–1.43	0.139	1.16	0.94–1.43	0.178
T stage						
T1	reference		** **	reference		
T2	1.15	0.77–1.56	0.447	1.29	0.84–1.75	<0.001
T3	1.46	1.08–1.97	0.05	1.6	1.14–2.12	<0.001
T4	2.16	1.43–3.28	0.007	1.89	1.55–3.68	<0.001
N stage						
N0	reference		** **	reference		
N1	1.10	0.80–1.65	0.603	1.21	1.68–2.6	0.3082
N2	1.46	1.01–2.12	0.013	1.55	1.74–2.52	0.0058
N3	1.55	1.33–2.68	<0.001	2.39	3.4–8.14	<0.001
Primary tumor surgery						
No	reference			reference		
Yes	0.57	0.40–0.70	<0.001	0.52	0.43–0.72	<0.001
Surgery to distant site						
No	reference		** **	reference		
Yes	0.89	0.65–1.20	0.430	0.78	0.56–1.09	0.149
Chemotherapy						
No/unknown	reference		** **	reference		
Yes	0.65	0.52–0.8	<0.001	0.49	0.35–0.73	<0.001
Radiotherapy						
No/Unknown	reference		** **	reference		
Yes	0.59	0.47–0.73	<0.001	0.64	0.51–0.81	0.001

PSM, propensity score matching; CSS, cancer specific survival; mHNC, metastatic head and neck cancer; HR, hazard ratio.

### A nomogram for identifying optimal candidates treated with PTS

**Figure 4:**
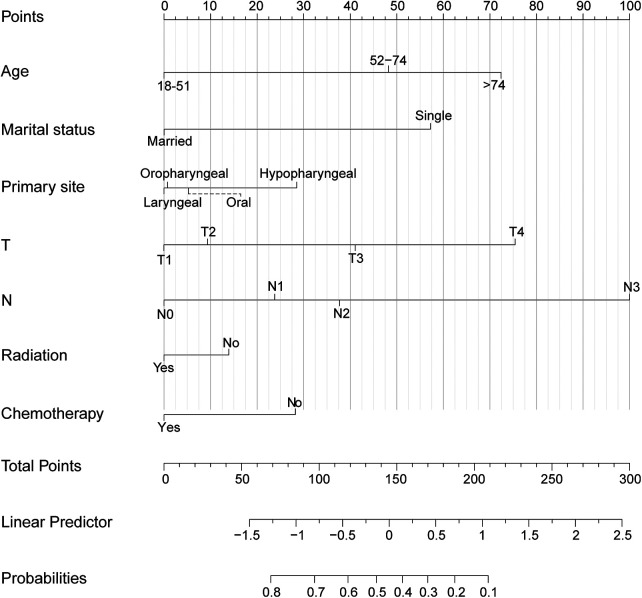
A nomogram for selecting the most suitable candidates for primary tumor excision.

The independent factors affecting CSS were included in the training set, comprising the TN stage, marital status, age, primary site, radiotherapy, and chemotherapy. Contingent on the findings from the multivariate logistic model analysis, we created the nomogram for the purpose of identifying patients with mHNC who would benefit significantly from surgical intervention at the primary site in the training group (Figure 4). The individual survival probability of patients was simply estimated by summing up the scores of each of the selected variables.

### Verification of the predictive model

**Figure 5:**
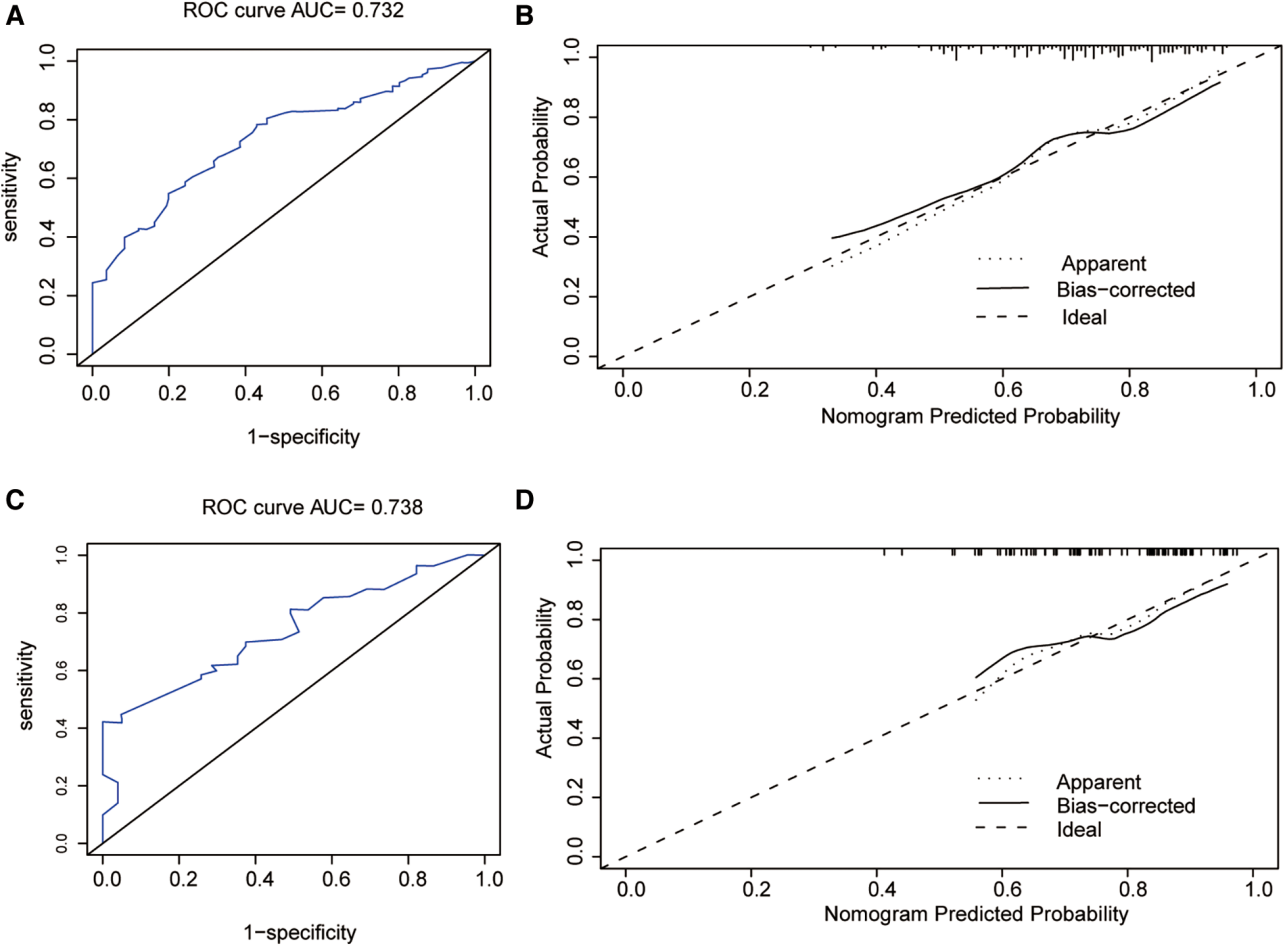
(**A**) Receiver operating characteristic curve of the nomogram in the training group. (**B**) The calibration plots of the training group. (**C**) Receiver operating characteristic curve of the nomogram in the validation group. (**D**) The calibration plots of the validation group. ROC, receiver operating characteristic.

The predictive nomogram had a good recognition in the training set (AUC = 0.732 CI 95% CI: 0.672–0.780) and the validation set (AUC = 0.738; 95% CI: 0.621–0.795). In addition, the correction curve showed that there was a strong correlation between the predicted value of the nomogram and the real observed value (Figure 5).

**Figure 6:**
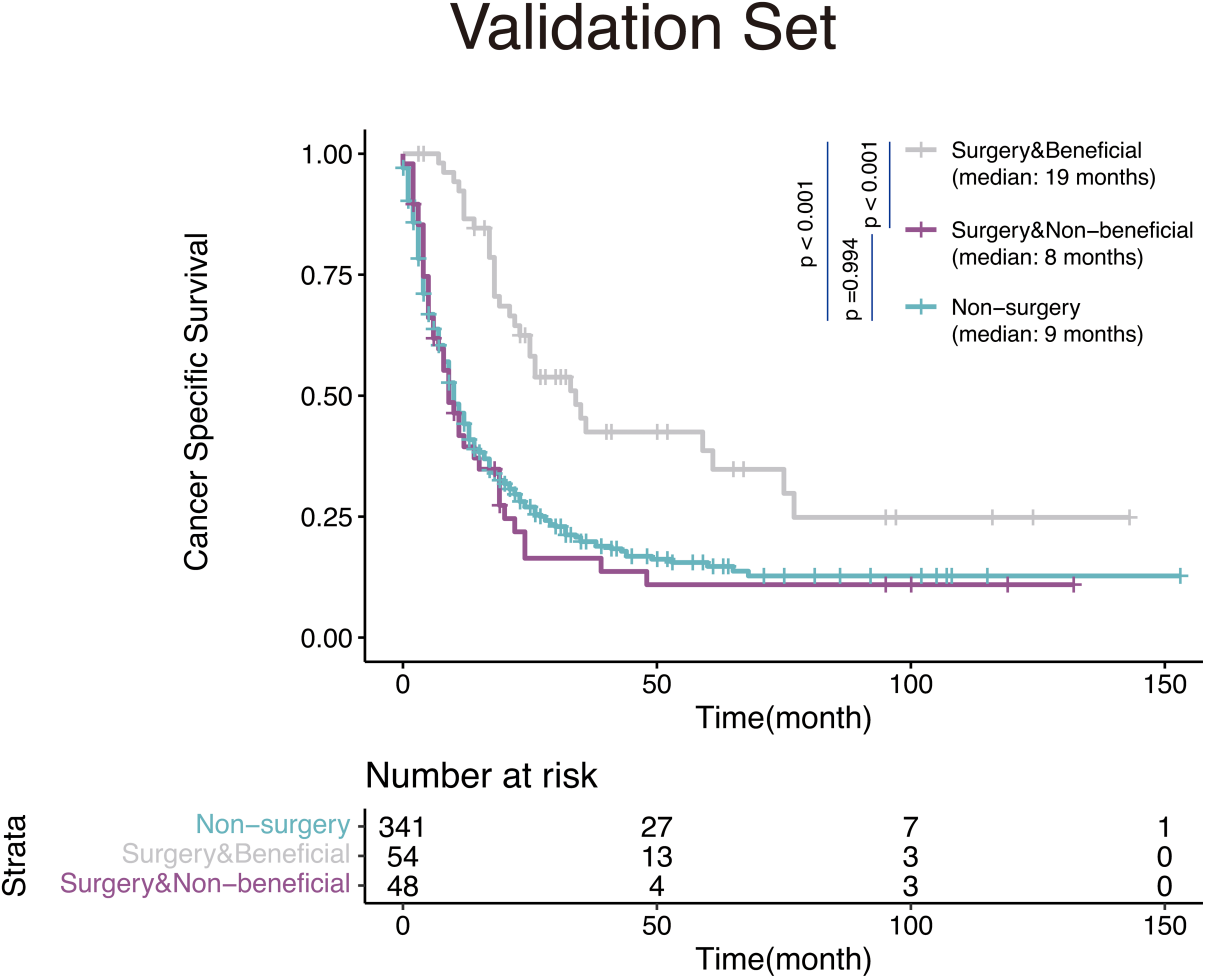
Kaplan-Meier survival curves for mHNC patients classified according to the nomogram-based distinct benefit categories.

We then validated the discrimination ability of the nomogram in the validation set. The Kaplan-Meier analysis and log-rank test as shown in Figure 6, demonstrated that the CSS in the surgery-beneficial group was significantly higher as opposed to that in the surgery-non-beneficial group (HR = 0.50, 95% CI: 0.35–0.68, *P* < 0.001) and the non-operation group (HR = 0.48, 95% CI,0.38–0.61, *P* < 0.001). No significant variations were identified between the surgery-non-beneficial and the non-PTS groups (HR = 0.99, 95% CI: 0.75–1.31, *P* = 0.994).

## Discussion

In this study, we found that PTS contributes to a significant survival advantage in patients with mHNC, suggesting that PTS has considerable potential for the treatment of mHNC. Subsequently, we developed a predictive nomogram to estimate the benefits of PTS. This study constructed a novel nomogram to identify patients with mHNC who had a greater likelihood of benefiting from PTS.

Although PTS is not a routine treatment option for metastatic cancer, several studies have confirmed its value for patients with metastatic cancer (21–23). Similar to the results of two previous studies, our study has shown that PTS improves the prognosis of patients with cancer (13, 14). Zumsteg et al. (13) focused on patients who received chemotherapy, while Patel et al. (14) did not rule out low-intensity palliative surgery such as photodynamic therapy, biopsy, and local tumor excision. In addition, to better maintain fundamental human function, PTS may destroy the seeding of primary tumor cells (24), reduce the production and release of tumor-associated growth factors and cytokines (25), and thus change the landscape of the immune microenvironment. This implies that PTS could play a role in reversing drug resistance and immunosuppression (26). Therefore, PTS may be a key link in the multimodality approach to improving the prognosis of patients with metastatic cancer.

In our predictive nomogram, the T stage, N stage, and age were the most important factors for predicting the efficacy of PTS in the mHNC patients, demonstrating that specific individual settings are crucial for selecting suitable patients for surgical treatment. For patients with an expected longer survival time and better individual conditions, aggressive local treatment may be beneficial. Therefore, it was relatively reasonable to choose mHNC patients undergoing the PTS who experienced a greater probability of benefiting from surgical intervention. Moreover, marital status is also an important predictor. Patients who are married may have a stronger desire for treatment and receive better care (27). In addition, radiotherapy and chemotherapy are also factors that cannot be ignored (12, 28). PTS combined with chemotherapy and/or radiotherapy may offer a favorable prognosis (13). This therapeutic option may be an effective treatment modality for patients with mHNC, but need further prospective trials are needed for validation.

The clinicopathological information varies greatly from patient to patient and the prognoses from case to case among mHNC patients. It is necessary to tailor the treatment regimens for different cases. So we developed a first population-based predictive nomogram to identify appropriate mHNC patients who benefit from PTS. The findings are expected to serve as a reference for mHNC patients to select PTS or not.

However, there are certain limitations to the present research. First, this was a retrospective analysis. Although we used PSM to eliminate bias, inherent bias from the database was unavoidable. Second, the lack of information about patient characteristics and complications in the SEER database made it impossible to assess the performance status of the patients, leading to treatment selection bias. Patients with favorable individual factors including better performance status and lower tumor burden were more likely to accept surgical intervention. However, surgery may have therapeutic effects masked by selection bias, and selection bias represents only some of the improved outcomes observed in patients with mHNC who underwent surgery. Third, a part of patients with “metastatic disease” when they have early primary/nodal stage (e.g., T1 N0) do not actually have distant disease. Often they may have cosynchronous primary (e.g., early stage lung cancer), which may lead to patient selection bias. However, inclusion of these patients in this analysis is reasonable as it highlights these patients should still receive surgery to the primary site when there is not complete clarity on the status of a potential metastatic site vs. cosynchronous primary. Finally, the SEER database did not contain some important information, such as systematic treatment plan, target therapies, site of metastasis, and the number of metastatic tumors, which also remarkably influence the patients' prognoses (29). Compared with patients with multiple distant metastasis, patients with oligometastasis may have a better prognosis (30). In addition, the treatment of metastatic sites may also prolong the survival time of patients, such as stereotactic body radiotherapy for pulmonary metastasis (31, 32). Despite these limitations, our study based on the SEER database does provide an additional and effective option for these patients. In further prospective trials, we will perform more investigations to confirm the validity of this model and improve upon it.

## Conclusions

In summary, we constructed a predictive model to identify the specific mHNC patients having a greater probability of benefiting from PTS. Patients with an age less than 52-years, well-differentiated tumors, T1, N0, and married individuals with mHNC may gain more benefit from PTS. Furthermore, this model needs to be validated in further prospective trials so as to benefit more patients.

## Data Availability

The original contributions presented in the study are included in the article/Supplementary Material, further inquiries can be directed to the corresponding author.
